# Safety, tolerability, and pharmacokinetics of repeated oral doses of 2-hydroxybenzylamine acetate in healthy volunteers: a double-blind, randomized, placebo-controlled clinical trial

**DOI:** 10.1186/s40360-020-0382-y

**Published:** 2020-01-06

**Authors:** Lisa M. Pitchford, Patricia M. Driver, John C. Fuller, Wendell S. Akers, Naji N. Abumrad, Venkataraman Amarnath, Ginger L. Milne, Sheau-Chiann Chen, Fei Ye, L. Jackson Roberts, M. Benjamin Shoemaker, John A. Oates, John A. Rathmacher, Olivier Boutaud

**Affiliations:** 1 MTI BioTech, Inc., Ames, IA 50010 USA; 2 Department of Kinesiology, Iowa State University, Ames, IA 50010 USA; 3 Department of Medicine, Division of Clinical Pharmacology, Vanderbilt University Medical Center, Nashville, TN 37232 USA; 4 Department of Pharmaceutical Sciences, Lipscomb University College of Pharmacy, Nashville, TN 37204 USA; 5 Department of Pharmacology, Division of Clinical Pharmacology, Vanderbilt University, Nashville, TN 37232 USA; 6 Department of Surgery, Vanderbilt University Medical Center, Nashville, TN 37232 USA; 7 Department of Biostatistics, Vanderbilt University Medical Center, Nashville, TN 37232 USA; 8 Department of Medicine, Division of Cardiology, Vanderbilt University Medical Center, Nashville, TN 37232 USA; 9 Department of Animal Science, Iowa State University, Ames, IA 50010 USA

**Keywords:** Safety, Pharmacokinetics, Humans, Salicylamine, γ-Ketoaldehydes

## Abstract

**Background:**

2-Hydroxybenzylamine (2-HOBA) is a selective dicarbonyl electrophile scavenger being developed as a nutritional supplement to help protect against the development of conditions associated with dicarbonyl electrophile formation, such as the cognitive decline observed with Mild Cognitive Impairment or Alzheimer’s disease.

**Methods:**

This study evaluated the safety, tolerability, and pharmacokinetics of repeated oral doses of 2-HOBA acetate (500 or 750 mg) administered to healthy volunteers every eight hours for two weeks. The effects of 2-HOBA on cyclooxygenase function and cerebrospinal fluid penetrance of 2-HOBA were also investigated.

**Results:**

Repeated oral administration of 2-HOBA was found to be safe and well-tolerated up to 750 mg TID for 15 days. 2-HOBA was absorbed within 2 h of administration, had a half-life of 2.10–3.27 h, and an accumulation ratio of 1.38–1.52. 2-HOBA did not interfere with cyclooxygenase function and was found to be present in cerebrospinal fluid 90 min after dosing.

**Conclusions:**

Repeated oral administration of 2-HOBA was found to be safe and well-tolerated. These results support continued development of 2-HOBA as a nutritional supplement.

**Trial registration:**

Studies are registered at ClinicalTrials.gov (NCT03555682 Registered 13 June 2018, NCT03554096 Registered 12 June 18).

## Background

Reactive dicarbonyl electrophiles, such as isolevuglandin, form in response to inflammation and oxidative stress, and have been implicated in the development and progression of many conditions. The dicarbonyl electrophiles react with lysine residues and form protein adducts capable of interrupting various cellular processes [1, 2]. Elevated levels of isolevuglandin-protein adducts have been observed in patients with a variety of clinical conditions, including, but not limited to, Alzheimer’s disease [3], atherosclerosis [4], hypertension [5], atrial fibrillation [6], and liver disease [7]. A mechanistic link exists between dicarbonyl electrophiles and Alzheimer’s disease development, as dicarbonyl electrophiles enhance the oligomerization and neurotoxicity of amyloid beta [8, 9].

2-HOBA can selectively and rapidly scavenge dicarbonyl electrophiles to prevent these dicarbonyl-associated protein modifications [10]. 2-HOBA is naturally occurring [11] and has been shown to be orally available in rodents and humans [12, 13]. In mice, 2-HOBA crosses the blood brain barrier, achieving 2-fold higher 2-HOBA levels in brain relative to plasma [12]. Critically, 2-HOBA administration protected hApoE4 mice from age-associated working memory deficits [14], suggesting a beneficial effect of 2-HOBA-mediated inhibition of dicarbonyl protein modification on the maintenance of hippocampal function.

In vitro and preclinical safety studies [15–18] did not indicate any toxicity concerns associated with 2-HOBA acetate within the expected therapeutic dose range. Similarly, the first-in-human study of 2-HOBA demonstrated excellent safety and tolerability in healthy volunteers at 2-HOBA acetate doses up to 825 mg^13^. In that study, 2-HOBA was rapidly absorbed with maximal plasma concentrations observed 1–2 h after oral administration, and was fully cleared from circulation between 8 and 24 h, suggesting dosing at least every 8 h would be required to maintain acceptable steady-state concentrations of 2-HOBA. The primary objective of the present investigation was to evaluate the safety and tolerability of three-times-daily dosing of 2-HOBA. Additional experiments were conducted to confirm 2-HOBA does not interfere with cyclooxygenase function and that that orally administered 2-HOBA can cross into the cerebrospinal fluid.

## Methods

The studies described herein are registered at ClinicalTrials.gov (multiple dose study - NCT03555682; cerebrospinal fluid penetrance study - NCT03554096). The study protocols were approved by the Vanderbilt University Institutional Review Board. All participants provided written informed consent before participating. All data was collected at the Vanderbilt University Medical Center Clinical Research Center between August 2018 and May 2019. This study and report adhere to CONSORT guidelines.

### Compound

2-HOBA was provided as the acetate salt (CAS 1206675–01-5). A commercial production lot produced under GMP (#16120312) of 2-HOBA acetate was obtained from TSI (China) Co., Ltd. (Shanghai, China) for use in this study. Our laboratory independently verified the purity of the commercial lot to be > 99% via HPLC and NMR spectroscopy. Hard gel capsules (Capsugel, Jiangsu, China) containing 250 mg of 2-HOBA acetate (corresponding to 168 mg 2-HOBA) were prepared by TSI (China) Co., Ltd. Fill weight, weight uniformity, disintegration, 2-HOBA content, acetate content, and microbial and analytical tests were within all specification limits.

### Subjects

Healthy male and non-pregnant female volunteers between the ages of 18 and 59 years were eligible to participate. Subjects were not permitted to take any medications 2 weeks prior to or during the study. Exclusion criteria included known cardiac, kidney, or hepatic disease; presence of diseases that could manifest morbidity or symptoms/signs that could confound interpretation of the study results; the need to discontinue any drug administered as standard of care treatment; and the unwillingness or inability to use approved birth-control methods.

### Multiple dose study design

This study was a double-blind, randomized, placebo-controlled, multiple dose escalation study designed to assess the safety, tolerability, and pharmacokinetics of multiple-dose 2-HOBA acetate. Nine subjects were studied per dose level, including 6 receiving 2-HOBA and 3 receiving placebo. Randomization was performed by the study statistician; a computer-generated randomization sequence using the stratified permuted block randomization, with blocks of size 3, was used to assign participants at the ratio of 1:2 to placebo or 2-HOBA at each dose level of the treatment. Treatments were provided by the Vanderbilt University Medical Center Pharmacy to study staff; participants and all study staff were blinded to treatment assignments. The dose levels of 2-HOBA acetate were 500 and 750 mg, which correspond to 336 and 504 mg 2-HOBA, respectively. 2-HOBA acetate was provided in 250-mg capsules; the placebo was identical in appearance and physical properties but contained no 2-HOBA acetate. These doses were designed to achieve peak plasma levels at steady state that approximate the peak plasma levels observed at the two highest doses used in the single dose study [13]. The dose frequency (every 8 h) was also based on the single dose pharmacokinetics [13]. Single doses of 2-HOBA acetate given to healthy volunteers resulted in 2-HOBA plasma concentrations in the range of 8.5–320 ng/ml at 8 h and no detectable levels 24 h following dose administration. Thus, an eight-hour dosing interval was selected for the multiple dose studies to ensure continued 2-HOBA exposure throughout the dosing interval.

On Day 1 of the study, volunteers were admitted to the clinical research center following an overnight fast, and baseline evaluations were performed, including vital signs (heart rate, respiration rate, blood pressure, and SpO2), clinical laboratory parameters (blood biochemistry, hematology, and urinalysis), and 12-lead ECG. The first dose of 2-HOBA acetate was administered with water, after which volunteers remained at the CRC for 24 h. Breakfast was provided after the baseline measurements were performed and the first dose of 2-HOBA was administered. Safety evaluations and potential adverse event assessments were performed at protocol-defined intervals. Volunteers were re-evaluated at the clinical research center briefly on day 5 and for 24 h following the last study dose on day 15. All adverse events were recorded, regardless of whether they were considered to be study-related. Additional follow-up evaluations were conducted by phone on days 3, 4, 8, and 11 of dosing as well as 3 and 7 days after discontinuing 2-HOBA acetate.

Blood samples for pharmacokinetic analyses were collected at baseline and 0.5, 1, 2, 4, 6, and 8 h after 2-HOBA acetate administration on days 1 and 15. An additional sample was collected 24 h after administration of the final dose of 2-HOBA. Urine samples (clean catch) were collected at baseline, 4, 8, 12 and 24 h after 2-HOBA acetate administration.

### Cerebrospinal fluid penetrance

A single oral dose of 2-HOBA acetate (550 mg) was administered to three volunteers (age 40–70 years). Ninety minutes after the dose administration, a lumbar puncture was performed to obtain cerebrospinal fluid and a blood sample was collected. 2-HOBA and salicylic acid levels were measured in cerebrospinal fluid and blood samples from this timepoint.

### Pharmacokinetic analysis

Plasma concentrations of 2-HOBA and the primary metabolite of 2-HOBA, salicylic acid, were determined for each timepoint in the multiple dose study and at a single timepoint in the cerebrospinal fluid penetrance study. 2-HOBA and salicylic acid were analyzed as described previously [13], with minor modifications. Briefly, standards, quality control samples, blanks, plasma, and cerebrospinal fluid were prepared for LC/MS/MS analysis by adding 100 μL of each to a protein precipitation filter plate containing acetonitrile and internal standard. Samples were eluted through the filter plate, and the eluent was dried down under nitrogen. Samples were reconstituted and sealed for analysis. LC-MS/MS analysis was performed on a Shimadzu LC coupled with a Sciex 6500 QTrap mass spectrometer (column: C18 50 × 2.1 mm, 1.7 μm, Phenomenex, Torrance, CA). The column temperature was set to 60 °C and the flow rate was 0.5 mL/min. A gradient of 3–90%B from 0 to 0.90 min was established by using a mobile phase A of 0.1% formic acid in water and mobile phase B of 0.1% formic acid in acetonitrile. 2-HOBA quantitation was performed in positive ionization mode (mass transition: 124.0 > 107.0), and salicylic acid quantitation was performed in negative ionization mode (mass transition: 137.1 > 93.1). Quantification of 2-HOBA was validated over the range of 5–5000 ng/mL, with within-run precision of 3.7–7.0%, bias − 9.7 **–** 2.8%, and between-run precision of 4.4–6.2%, bias − 7.1 – 1.6% [13]. In-process analytical performance of 2-HOBA during routine analysis of samples demonstrated an intra-assay precision of 1.1–14.8%, bias − 4.0 **–** 17.1%, and inter-assay precision of 3.7–9.0%, bias 6.0–9.0%. Quantification of salicylic acid in samples was qualified over the range of 100–5000 ng/mL. In-process analytical performance of salicylic acid during routine analysis of samples demonstrated an intra-assay precision of 2.3–8.8%, bias − 5.2 **–** 8.7% and inter-assay precision of 4.6–6.4%, bias − 1.5 **–** 6.2%. All standards and quality control samples for 2-HOBA and salicylic acid met acceptance criteria (standard curve R^2^ > 0.90, 66.7% of all QC samples and at least 50% at each concentration within 15% of nominal concentration).

Plasma concentration-time data were imported into Phoenix WinNonlin® 8.0 software (Certara USA, Inc., Princeton, NJ) to estimate the oral pharmacokinetic parameters of 2-HOBA in individual subjects. Noncompartmental analysis using Model 200 (Plasma; Single Extravascular Dose; Linear Log Trapezoidal Method) was performed on each plasma concentration-time profile to estimate the following individual pharmacokinetic parameters: elimination rate constant (K_e_), elimination half-life, apparent volume of distribution (V_d_/F), apparent clearance (Cl/F), area under the concentration-time curve (AUC), maximum observed plasma concentration (C_max_), the time to reach the maximum observed plasma concentration (T_max_), and the accumulation index from Day 1 to Day 15. The accumulation index for 2-HOBA was calculated by taking the ratio of the AUC_0–8h_ on Day 15 (last dose) relative to the AUC_0–8h_ on Day 1 (first dose). The average 2-HOBA concentration (C_avg_) during the dosing interval on Day 1 and Day 15 was calculated by dividing the AUC_0–8h_ of the first dose and last dose by the dosing interval. The percent peak-to-trough fluctuation (%PTF) during the dosing interval on Day 1 and Day 15 was calculated by subtracting the minimum 2-HOBA concentration (C_min_) from the C_max_ divided by their respective C_avg_. In addition to calculating the elimination half-life from the elimination rate constant, an effective half-life (t_½eff_) was calculated based on both the dosing interval and accumulation index from Day 1 to Day 15 following multiple dose administration using the following equation [19, 20]:

$$ {t}_{\frac{1}{2} eff}=\frac{Dosing\ Interval\ast \mathit{\ln}2}{\ln \left(\frac{Accumulation\ Index}{Accumulation\ Index-1}\right)} $$

### Urinary prostaglandin metabolites analysis

To assess whether the major 2-HOBA metabolite, salicylic acid, inhibited cyclooxygenases during the study, concentrations of the urinary metabolites of prostaglandin E2 (PGE-M), thromboxane B2 (TxB2-M), and prostacyclin (PGI-M) in urine were measured in the Eicosanoid Core Laboratory at Vanderbilt University Medical Center. Urine (1 mL) collected at baseline on day 1 (before 2-HOBA acetate administration) and before the last dose on day 15, was acidified to pH 3 with HCl. [^2^H_4_]-2,3-dinor-6-keto-PGF1α (internal standard for PGI-M quantification), and [^2^H_4_]-11-dehydro-TxB_2_ were added, and the sample was treated with methyloxime HCl to convert analytes to the *O*-methyloxime derivative. The derivatized analytes were extracted using a C-18 Sep-Pak (Waters Corp. Milford, MA USA) and eluted with ethyl acetate as previously described [21]. A [^2^H_6_]-*O*-methyloxime PGE-M deuterated internal standard was then added for PGE-M quantification. The sample was dried under a stream of dry nitrogen at 37 °C and then reconstituted in 75 μL mobile phase A (see below) for LC/MS analysis.

LC was performed on a 2.0 × 50 mm, 1.7 μm particle Acquity BEH C18 column (Waters Corporation, Milford, MA, USA) using a Waters Acquity UPLC. Mobile phase A was 95:4.9:0.1 (v/v/v) 5 mM ammonium acetate:acetonitrile:acetic acid, and mobile phase B was 10.0:89.9:0.1 (v/v/v) 5 mM ammonium acetate:acetonitrile:acetic acid. Samples were separated by a gradient of 85–5% of mobile phase A over 14 min at a flow rate of 375 μl/min prior to delivery to a SCIEX 6500+ QTrap mass spectrometer. Urinary creatinine levels were measured using a test kit (Enzo Life Sciences, Farmingdale, NY, USA). The urinary metabolite levels in each sample were normalized to the urinary creatinine level of the sample and expressed in ng/mg creatinine.

### Statistical analyses

Descriptive statistics (means, standard deviations, standard error for continuous data, frequency and percentage for categorical data, etc.) were used to summarize demographics, safety, pharmacokinetic assessments, and prostaglandin metabolite assessments. Kruskal-Wallis test for continuous data and Pearson’s chi-squared test for categorical data were used to test for group differences in demographic characteristics. Pre- vs. post- 2-HOBA treatment differences across the three dose levels (placebo, 500 mg, and 750 mg) were assessed for each urinary prostaglandin metabolite using the mixed-effect model to take into account the correlation structure with the repeated measures data. Using model-based (least-square) means, the average adjusted change from pre- and post-treatment for each dose level and the placebo group were estimated and compared using Wald test. Bonferroni correction was used to adjust for multiple comparisons. Standardized residual analysis was performed to evaluate model assumptions. In order to improve the data distribution, PGE-M was analyzed after natural log transformation (TxB2-M and PGI-M were analyzed on the original scale). Study data were collected and managed using REDCap electronic data capture tools hosted at Vanderbilt University [22].

## Results

### Multiple dose study

A total of 18 volunteers were enrolled in the multiple dose study (6 volunteers at each dose level and 6 placebo). Subject demographics are provided in Table 1. There were no significant differences in demographic characteristics between treated and placebo subjects or between dose groups.

**Table 1:** Demographic Characteristics

	Placebo	500 mg 2-HOBA acetate	750 mg 2-HOBA acetate	Total
Volunteers (*n*)	6	6	6	18
Sex: female [*n* (%)]	4 (67)	3 (50)	4 (67)	11 (61)
Age (y)	33.5 ± 13.6	35.2 ± 13.8	30.8 ± 11.9	33.2 ± 12.5
Height (cm)	167.6 ± 11.0	175.3 ± 11.6	169.3 ± 9.0	170.8 ± 10.6
Weight (kg)	64.2 ± 10.3	76.3 ± 9.7	70.7 ± 9.4	70.4 ± 10.6
BMI (kg/m^2^)	22.8 ± 1.8	24.8 ± 2.5	24.6 ± 2.2	24.0 ± 2.3
Race [*n* (%)]				
Black/African American	1 (17)	2 (33)	0 (0)	3 (17)
White	5 (83)	4 (67)	6 (100)	15 (83)
Ethnicity [*n* (%)]				
Hispanic/Latino	1 (17)	1 (17)	0 (0)	2 (11)
Not Hispanic/Latino	4 (80)	5 (83)	6 (100)	15 (88)
Not Reported	1 (17)	0 (0)	0 (0)	1 (6)

Data are presented as means ± SD unless otherwise noted

No serious or severe adverse events or deaths were observed. All reported adverse events are summarized in Table 2. Fourteen participants (78%) reported at least 1 adverse event during the study. The most common reported adverse event was headache (6 subjects, 33%). All adverse events were mild in intensity and transient, with the exception of one volunteer that experienced a rash defined as moderate in intensity; this volunteer was treated for the rash and withdrawn from the study. No adverse events were determined to be study-related, and neither adverse event frequency nor severity were dose-dependent. These adverse events were reported and reviewed by a National Institute of Aging (NIA) approved Data and Safety Monitoring Board (DSMB). No clinically significant changes in ECG recordings, vital signs, or laboratory parameters that were considered to be related to 2-HOBA were observed.

**Table 2:** Summary of reported adverse events by dose

	2-Hydroxybenzylamine acetate dose			Total (*n* = 18)
	Placebo (*n* = 6)	500 mg (*n* = 6)	750 mg (*n* = 6)	
Any event, n (%)	4 (67)	6 (100)	4 (67)	14 (78)
Headache	2 (33)	2 (33)	2 (33)	6 (33)
GI distress (nausea, bloating, constipation)	2 (33)	1 (17)	0 (0)	3 (17)
Rash/itching	1 (17)	1 (17)	1 (17)	3 (17)
Urine odor	0 (0)	2 (33)	0 (0)	2 (11)
Dry mouth	1 (17)	1 (17)	0 (0)	2 (11)
Nasal congestion	0 (0)	2 (33)	0 (0)	2 (11)
Lethargy/sleepiness	0 (0)	1 (17)	1 (17)	2 (11)
Hypertension	0 (0)	1 (17)	0 (0)	1 (6)
Eye irritation	0 (0)	1 (17)	0 (0)	1 (6)

**Fig. 1:**
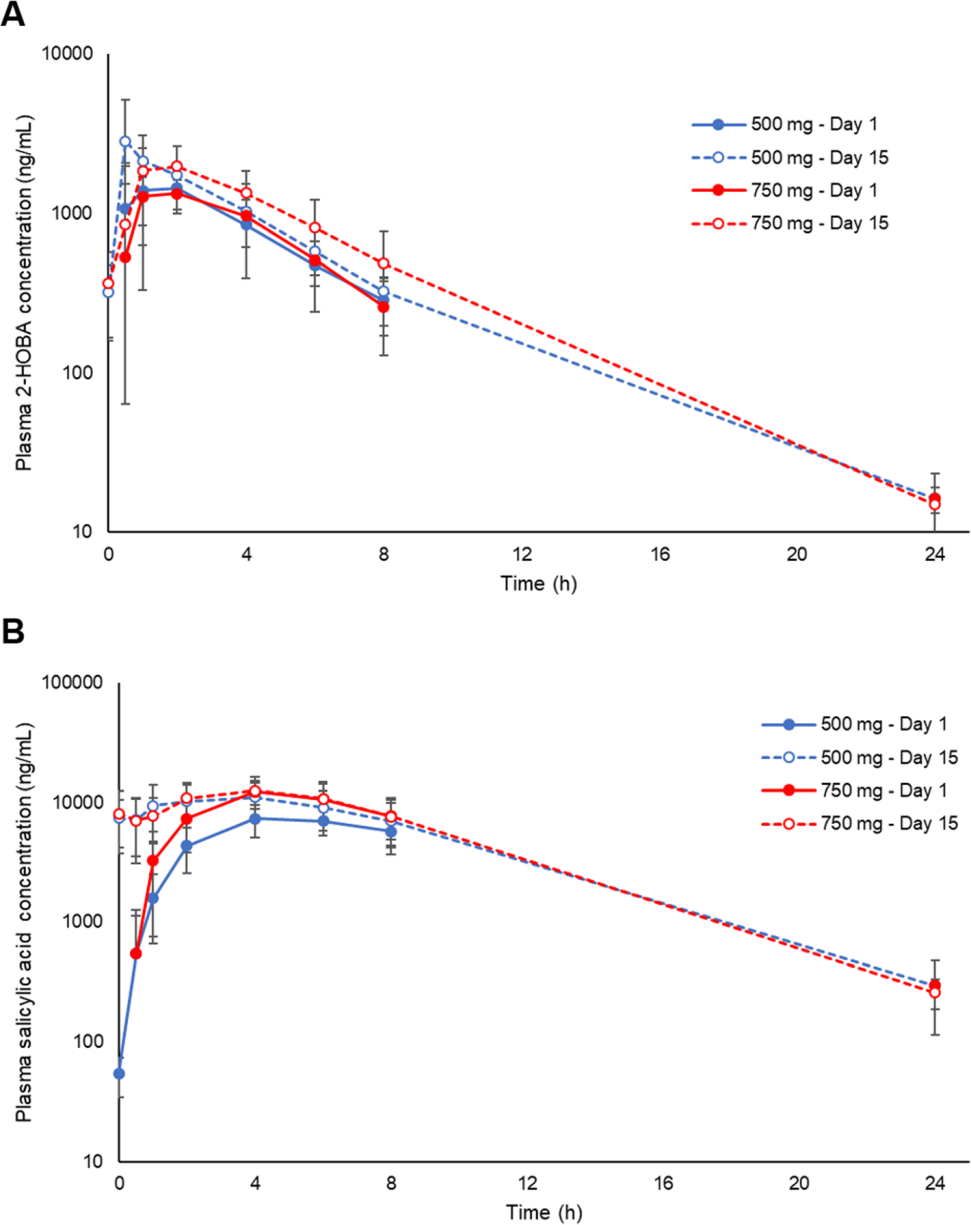
2-Hydroxybenzylamine (2-HOBA) and primary metabolite (salicylic acid) plasma concentrations after oral administration of 2-HOBA acetate. Plasma concentrations of 2-HOBA (**a**) and salicylic acid (**b**) were measured for 8 (first dose) or 24 (last dose) hours after oral administration of 2-HOBA acetate at two dose levels

Mean 2-HOBA plasma concentration-time profiles are shown in Fig. 1a, and pharmacokinetic parameter estimates are presented in Table 3. No 2-HOBA was detectable prior to administration on day 1 in any subjects or during the study for any subject administered the placebo treatment. 2-HOBA was rapidly absorbed at both dose levels with an average T_max_ that ranged between 0.8–2 h. Pharmacokinetic 2-HOBA exposure parameters (C_max_ and AUC) and half-life were similar between dose groups, with a higher C_max_ observed on Day 15 in the 500 mg dose group largely driven by a high 2-HOBA C_max_ in one volunteer (7047 ng/ml at 0.5 h). Estimates of 2-HOBA clearance and volume of distribution following oral administration tended to be higher in the high dose group compared to the low dose group. Pharmacokinetic parameters (half-life, volume of distribution, clearance) following the last dose were similar to the first dose in both treatment groups; whereas, 2-HOBA exposure (C_max_ and AUC) increased from the first dose to the last dose. Increased 2-HOBA exposure at steady-state for the low dose group and the high dose group was associated with an accumulation index of 1.38 and 1.52, respectively. The accumulation index relative to the 8-h dosing interval resulted in a t_½eff_ of 4.26 ± 0.93 h in the low dose group and 5.15 ± 1.64 h in the high dose group.

**Table 3:** Mean 2-hydroxybenzylamine pharmacokinetic parameters after oral doses

Parameter	2-Hydroxybenzylamine acetate dose			
	500 mg (*n* = 6)		750 mg (*n* = 5–6)	
	Day 1 (first dose)	Day 15 (last dose)	Day 1 (first dose)	Day 15 (last dose)
K_e_	0.28 ± 0.04	0.22 ± 0.05	0.34 ± 0.06	0.22 ± 0.01
Half-life (h)	2.53 ± 0.39	3.27 ± 0.61	2.10 ± 0.45	3.12 ± 0.08
V_d_/F	162 ± 21	183 ± 58	225 ± 79	264 ± 85
Cl/F	44.9 ± 6.3	38.2 ± 6.7	73.9 ± 18.5	58.9 ± 19.9
T_max_ (h)	1.17 ± 0.68	0.83 ± 0.61	1.75 ± 1.25	2.00 ± 1.22
C_max_ (ng/mL)	1955 ± 422	3177 ± 1993	1916 ± 524	2292 ± 913
C_avg_ (ng/mL)	817 ± 88	1133 ± 225	798 ± 193	1190 ± 455
C_min_ (ng/mL)	285 ± 113	324 ± 50	261 ± 133	484 ± 287
PTF (%)	207 ± 67	237 ± 110	215 ± 87	158 ± 58
AUC_0–8h_ (h·ng/mL)	6536 ± 701	9063 ± 1796	6385 ± 1547	9523 ± 3638
AUC_inf_ (h·ng/mL)	7620 ± 1230	10,605 ± 1740	7186 ± 1780	11,746 ± 4602
Accumulation Index		1.38 ± 0.15		1.52 ± 0.27

K_e_, elimination rate constant; V_d_/F, apparent volume of distribution; Cl/F, apparent clearance; T_max_, time to reach maximum plasma concentration; C_max_, maximum observed plasma concentration; C_avg_, average observed plasma concentration; C_min_, minimum observed plasma concentration; PTF, peak-to-trough fluctuation; AUC_0–8_, area under the dosing interval curve; AUC_inf_, area under the concentration-time curve from zero to infinity.

Plasma concentrations of the primary metabolite of 2-HOBA, salicylic acid, were also measured in each subject. Mean 2-HOBA plasma concentration-time profiles are shown in Fig. 1b, and pharmacokinetic parameter estimates are presented in Table 4. Salicylic acid exposure (e.g. C_max_, AUC) tended to be higher after the first dose of 750 mg 2-HOBA compared to 500 mg 2-HOBA, but parameters were similar after the multiple dosing regimen. Visual examination of salicylic acid relative to 2-HOBA concentrations versus time on a semi-log plot suggests that the metabolite exhibits formation rate-limited disposition kinetics (data not shown).

**Table 4:** Mean salicylic acid pharmacokinetic parameters after oral doses of 2-hydroxybenzylamine

Parameter	2-Hydroxybenzylamine acetate dose			
	500 mg (*n* = 6)		750 mg (*n* = 5–6)	
	Day 1 (first dose)	Day 15 (last dose)	Day 1 (first dose)	Day 15 (last dose)
T_max_ (h)	5.00 ± 1.10	3.50 ± 1.22	4.67 ± 1.03	3.20 ± 1.79
C_max_ (ng/mL)	7635 ± 2112	11,382 ± 4118	12,092 ± 2619	12,768 ± 3703
AUC_0–8h_ (h·ng/mL)	42,458 ± 12,070	75,288 ± 28,095	65,935 ± 13,775	81,826 ± 28,120
AUC (h·ng/mL)		123,075 ± 55,044		117,842 ± 41,615

C_max_, maximum observed plasma concentration; T_max_, time to reach C_max_; AUC, area under the concentration-time curve from zero to infinity; AUC_0–8_, area under the dosing interval curve.

**Fig. 2:**
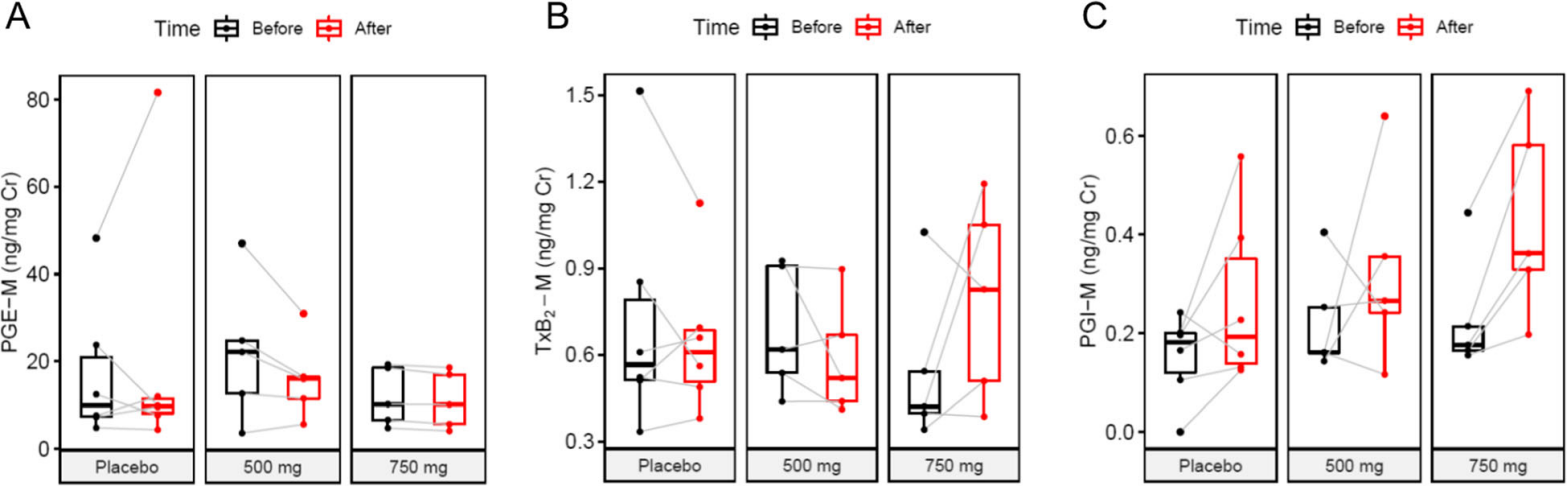
2-Hydroxybenzylamine (2-HOBA) metabolite does not inhibit cyclooxygenases. Urinary metabolites of **a**) prostaglandin E2 (PGE-M), **b**) thromboxane B2 (TxB2-M), and **c**) prostacyclin (PGI-M) were measured by LC-ESI/MS/MS before and after treatment. There were no significant changes in urinary metabolite concentration (mixed-effects model, *n* ≥ 5)

Urinary prostaglandin metabolites were measured to determine whether salicylic acid accumulation over the multiple 2-HOBA dosing regimen inhibited the cyclooxygenases. The metabolites of all three major prostaglandins indicated no significant difference between baseline pre-dosing and at the end of the study for placebo or either 2-HOBA dose (Fig. 2).

### Cerebrospinal fluid penetrance

Two male and one female volunteers (age 50 ± 9), completed the cerebrospinal fluid penetrance study. 2-HOBA was detectable in cerebrospinal fluid in all three volunteers at an average concentration of 462 ± 327 ng/ml. These values correspond to 34–74% of the concentration observed in plasma from the same timepoint. In contrast, the primary metabolite, salicylic acid, was also detectable with an average concentration of 78.2 ± 76.8 ng/ml, which corresponded to 0.5–1.7% of the observed plasma concentration.

## Discussion

Multiple doses of 2-HOBA acetate (up to 750 mg TID) were well tolerated by healthy individuals in this study. No serious adverse events were observed, and none of the reported adverse events were determined to be related to 2-HOBA. 2-HOBA acetate was not associated with any clinically significant findings in vital signs, ECG recordings, or clinical laboratory parameters.

The pharmacokinetic properties of 2-HOBA observed in this study are generally similar to those observed previously in mice [12] and humans [13]. As with single doses of 2-HOBA acetate [13], 2-HOBA was readily absorbed with a T_max_ of 1 to 2 h in the multiple dose study groups and achieved steady-state maximal 2-HOBA concentrations in the range of those observed with the highest doses in the previous single ascending dose study. The AUC increased from day 1 to day 15, indicating 2-HOBA accumulation with both 2-HOBA acetate dosing regimens, yielding accumulation ratios ranging from 1.19–1.94. The greater accumulation of 2-HOBA in the current multiple dose study was higher than predicted (1.06–1.22) in the previous single ascending dose study [13]. Underprediction of 2-HOBA accumulation can be attributed, in part, to a slightly longer estimated elimination half-life and an even longer effective half-life relative to the dosing interval used in the present study. The utility in calculating an effective half-life to better predict accumulation with multiple dosing strategies and modified dosage formulations has highlighted the importance of accounting for dosing regimen factors (route of administration, dose, and dosing interval) and unknown or complex disposition processes (absorption, distribution, and elimination) that impact overall exposure. The greater than predicted accumulation index in the current multiple dosing study further substantiates that dosing every 8 h may be sufficient to maintain 2-HOBA exposure throughout the dosing interval.

Unlike the dose-dependent increase in 2-HOBA exposure observed across a broader single dose range [13], average systemic exposure (C_max_ and AUC) to 2-HOBA was similar for 500 and 750 mg doses. This may be related to considerable inter-subject and inter-study variability in response to 2-HOBA oral administration, as the 500 mg dose resulted in greater exposure on average than was observed in the previous single-dose study. In addition, oral bioavailability of 2-HOBA has not yet been established in humans and may vary considerably based on dose, the gastrointestinal environment, processes regulating its absorption, concomitant medications, and other unknown individual-specific factors. Thus, the lack of a dose-dependent increase in 2-HOBA exposure in this study could be attributed to an unknown factor that increased bioavailability in the low dose group or decreased bioavailability in the high dose group. This unknown bioavailability in both the low and high dose groups limits the interpretation of both clearance and volume of distribution following oral administration of 2-HOBA.

The major metabolite of 2-HOBA was determined to be salicylic acid [17] most likely through a amine oxidase biotransformation [17, 23, 24]. Peak plasma concentration of salicylic acid at the high dose was 12.8 ± 3.7 mg/L, a concentration well below the accepted anti-inflammatory therapeutic range (150–300 mg/L) [25]. Importantly, our data shows that 2-HOBA administration at either dose, does not significantly inhibit the cyclooxygenases, as reflected by measuring the urinary metabolites of the 3 major prostaglandins, PGE-M, TxB_2_-M and PGI-M.

Because measuring brain tissue levels is not possible in living humans, we compared 2-HOBA levels in the plasma to those in the cerebrospinal fluid of three volunteers who took a single dose of 2-HOBA. Our results show that cerebrospinal fluid levels were between 34 to 74% of those in plasma. Our results also demonstrate that salicylic acid in the cerebrospinal fluid represented only 0.5–1.7% relative to plasma. The very high plasma protein binding of salicylic acid relative to 2-HOBA was one factor that contributed to higher levels of 2-HOBA in cerebrospinal fluid compared to salicylic acid [17, 26]. However, both compounds also demonstrate a high degree of ionization at a physiologic pH of 7.4, which could increase the time required to reach distribution equilibrium in CSF. Predicted pKa values for the carboxylic acid group on 2-HOBA (parent) and the primary amine group on salicylic acid (metabolite) are estimated to be 2.79 and 8.63, respectively. As such, both compounds would be almost completely ionized in the systemic circulation with only ~ 5% difference in the unionized species in favor of 2-HOBA. In addition to plasma protein binding and percent ionization, substrate specificity for efflux transporters at the blood-CSF barrier could also play an important role in establishing equilibrium concentrations in the CSF. Although cerebrospinal fluid compound levels do not always reflect brain tissue levels [27], our data combined with the favorable chemical structure of 2-HOBA and the observation that 2-HOBA crosses the blood brain barrier in mice [12] suggest that 2-HOBA passes the blood brain barrier in humans.

Together, these observations continue to support the tolerability and safety of 2-HOBA in humans and add further support to a growing portfolio of preclinical and early clinical safety data [13, 15–18]. This portfolio, combined with the preclinical efficacy established in mice at risk for age-related cognitive decline [14] support continued development of 2-HOBA as a nutritional supplement to enhance cognitive health and support healthy brain aging.

As this study was conducted with a small number of healthy volunteers, the generalizability of the results is limited. Tolerability studies of 2-HOBA acetate should be conducted in additional populations, such as older adults and/or adults with chronic disease to allow for the identification of any unique adverse effects or pharmacokinetic properties in these populations. Additionally, future work is required to evaluate the pharmacological and physiological effects of 2-HOBA in humans.

## Conclusions

2-HOBA acetate was found to be safe and well-tolerated at dose regimens up to 750 mg TID in healthy human volunteers. The pharmacokinetic profile demonstrated that 2-HOBA was detected throughout the 8 h dosing interval and that plasma concentrations at steady-state accumulated approximately 40 to 50% following multiple doses. The next steps for development include evaluating the safety and tolerability of multiple doses of 2-HOBA acetate in older individuals, who better represent the target population of individuals with elevated risk of developing Alzheimer’s disease.

## Data Availability

The data for this research study were collected under informed consent of the volunteers. Thus, access to the data will be subject to approval by the Vanderbilt University Medical Center Institutional Review Board.

## Abbreviations

%PTF: Percent peak-to-trough fluctuation
2-HOBA: 2-hydroxybenzylamine
AUC: Area under the concentration-time curve
BMI: Body mass index
C_avg_: Average observed plasma concentration
Cl/F: Apparent clearance
C_max_: Maximum observed plasma concentration
C_min_: Minimum observed plasma concentration
DSMB: Data and Safety Monitoring Board
HPLC: High-performance liquid chromatography
K_e_: Elimination rate constant
NIA: National Institute on Aging
NMR: Nuclear magnetic resonance
PGE-M: Urinary metabolite of prostaglandin E2
PGI-M: Urinary metabolite of prostacyclin
t_½eff_: Effective half-life
t_1/2_: Half-life
TID: Three times daily
T_max_: Time to reach maximum observed plasma concentration
TxB2-M: Urinary metabolite of thromboxane B2
V_d_/F: Apparent volume of distribution
